# Effectiveness of aquatic Tai Chi on balance indicators: a systematic review

**DOI:** 10.3389/fpubh.2026.1774853

**Published:** 2026-03-30

**Authors:** Grzegorz Mańko, Tadeusz Ambroży, Krzysztof Kasicki, Jarosław Jaszczur-Nowicki, Mariusz Ozimek, Piotr Czech, Paweł Gąsior, Łukasz Rydzik

**Affiliations:** 1 Department of Biomechanics and Kinesiology, Institute of Physiotherapy, Jagiellonian University Collegium Medicum, Kraków, Poland; 2 Małopolska Rehabilitation Hospital in Krzeszowice, Kraków, Poland; 3 Department of Sport Theory and Motor Skills, Institute of Sports Sciences, University of Physical Culture in Kraków, Kraków, Poland; 4 Department Physiotherapy, School of Public Health, Collegium Medicum, University of Warmia and Mazury, Olsztyn, Poland; 5 Faculty of Physical Culture and Safety Sciences, Academy of Applied Sciences in Nowy Sącz, Nowy Sącz, Poland; 6 Institute of Humanities and Tourism, Academy of Applied Sciences in Nowy Targ, Nowy Targ, Poland

**Keywords:** aging, aqua therapy, older adults, exercise, fall risk, well being

## Abstract

**Background:**

Balance disorders expose older adults to an increased risk of falls. The combination of Tai Chi and an aquatic environment may improve balance. The aim of this review was to assess the effectiveness of Ai Chi in improving balance in older adults.

**Methods:**

The systematic review was conducted in accordance with the PRISMA guidelines. Criteria: population individuals aged ≥60 years with balance disorders or at risk of falling; intervention Ai Chi (Tai Chi in water); control no intervention or other exercises; outcomes balance measures. The PubMed, Scopus, Web of Science and EBSCO databases were searched without time restrictions for RCTs and quasi experimental studies. Study selection and risk-of-bias assessment were performed independently by two authors.

**Results:**

Seven studies (6 RCTs, 1 non-randomized) with a total of 213 participants were included. Ai Chi programs lasted from 6 to 14 weeks (1–3 sessions/week). All studies showed an improvement in balance in the Ai Chi groups compared with controls. A significant improvement in static and dynamic balance (shortening of TUG time) was observed in the Ai Chi groups. In one study, only Ai Chi participants achieved a TUG score below the fall-risk threshold. Ai Chi proved to be as effective as land-based exercise and aquatic kinesiotherapy. No serious adverse events were reported.

**Conclusion:**

Ai Chi effectively improves balance in older adults, which may reduce the risk of falls and improve mobility. It is a safe, low joint-load form of exercise recommended as an adjunct to geriatric physiotherapy. A limitation is the small number of studies of moderate quality. Further large-scale RCTs with longer follow-up are needed to confirm the long-term effects of Ai Chi.

**Systematic review registration:**

PROSPERO, Identifier (CRD420251162371).

## 1 Introduction

Population ageing is one of the most serious demographic and health challenges of the 21st century. According to estimates by the World Health Organization (WHO), by 2050 the population of people aged 60 years and older will double, reaching 2.1 billion (1). With age, there is progressive involution of physiological systems crucial for maintaining postural control, including the vestibular, visual and somatosensory systems (2). This process, combined with sarcopenia and impaired neuromuscular function, drastically increases the risk of falls (3). Many older adults also experience fear of falling, which affects approximately 30% of people over 60 years of age (4). The problem reaches much deeper and has a significant impact on public healthcare finances, making it one of the most important issues in the contemporary population (5).

Current clinical guidelines clearly indicate physical activity as the most effective method of fall prevention (6). Among the recommended interventions, Tai Chi, a form of mind–body training, holds a special position (7). Numerous meta-analyses have confirmed the effectiveness of land-based Tai Chi in improving postural control, coordination and reducing the risk of falls in older adults and in populations with neurological disorders such as Parkinson’s disease (8, 9). However, for part of the geriatric population, especially individuals with advanced degenerative joint changes, obesity or significant motor impairment, land-based exercises may be too demanding or associated with a risk of injury (10).

The combination of Tai Chi principles with the specificity of the aquatic environment led to the development of the Ai Chi method. Ai Chi is a sequence of slow, flowing movements of the upper and lower limbs and trunk, coordinated with deep diaphragmatic breathing, performed in water at shoulder depth (11). This method integrates training of centre of mass control over the base of support with elements of relaxation and concentration (12). In recent years, there has been growing research interest in this form of therapy.

A solution to this problem has been the combination of exercise with the aquatic environment. Water creates specific unloading and simultaneously neuromuscularly stimulating conditions (13). Buoyant force reduces effective body weight, unloading the joints and decreasing the risk of pain during exercise (14–16). Hydrostatic pressure supports venous return and reduces oedema, while the viscosity of water provides natural resistance, enhancing proprioceptive feedback (17). Crucial for balance training, the aquatic environment increases the reaction time to loss of stability, giving the patient more time to correct posture, while at the same time eliminating the risk of injury in the event of a fall, which reduces fear and encourages the undertaking of more challenging motor tasks (18).

Despite the growing number of publications, evidence on the effectiveness of Ai Chi compared to standard land-based interventions or other forms of hydrotherapy remains inconclusive. Some studies indicate the superiority of Ai Chi over land-based therapies in terms of improving dynamic balance and reducing bradykinesia (19), whereas others report no significant between-group differences (20, 21). Existing systematic reviews often combine Ai Chi with other forms of aquatic exercise, which makes it difficult to isolate the specific effect of this method (22). Moreover, few studies synthesise results using clinically validated balance indicators in populations aged around 60 years and older, including both healthy individuals and those with neurological deficits.

The primary aim of this systematic review was to evaluate the effectiveness of Ai Chi interventions in improving balance parameters in older adults. The specific objectives of the study were: to determine the effect of Ai Chi on static balance, to assess its impact on dynamic balance and mobility, and to conduct a comparative analysis of the effectiveness of Ai Chi versus conventional land-based exercises and other forms of hydrotherapy in individuals aged ≥60 years. Based on these objectives, an analysis was conducted and an overall outline is presented below in light of the discussed issues and the existing literature.

Based on the current state of clinical knowledge, the following research hypotheses were formulated:

1) Ai Chi–based interventions lead to improvements in static and dynamic balance parameters in older adults compared with the absence of targeted physical activity.
2) Aquatic training in the form of Ai Chi demonstrates superior or at least comparable effectiveness in reducing fall risk compared with standard land-based balance exercises.
3) The application of the Ai Chi method yields greater functional benefits in the domain of postural stability than other general hydrotherapy interventions.

## 2 Methods

The systematic review was carried out in accordance with the Preferred Reporting Items for Systematic Reviews and Meta-Analyses (PRISMA) guidelines (23). The assumptions and protocol of the review were developed in advance and registered *a priori* in the PROSPERO database under the number CRD420251162371 and are available online. The authors of the review do not derive any financial benefits from the results concerning the methods in question and are not affiliated with any rehabilitation method presented in the review.

### 2.1 Eligibility criteria (PICOs framework)

The PICOs criteria were applied to exclude specific types of scientific publications and to select articles with a high scientific standard (Table 1).

**Table 1:** PICOs Inclusion and exclusion criteria.

	Inclusion criteria	Exclusion criteria
P	- Older adults (≥60 years); trials with mixed ages are eligible if mean age ≥60 or ≥75% of participants are ≥60, or if older-adult subgroup data are extractable. - With or without balance impairment or history of falls; stable chronic conditions allowed if medically cleared for exercise and aquatic activity.	- Severe cognitive impairment that prevents informed participation. - Non-ambulatory individuals requiring full physical assistance for transfers/locomotion.
I	- Tai Chi performed in water (aquatic Tai Chi / Ai Chi; acceptable forms declared as “water-based Tai Chi”). - Acceptable standard co-interventions identical in both arms (such as education). - No restrictions regarding water depth/temperature or Tai Chi style (reported as potential moderators).	- Mixed programs in which Tai Chi accounts for <70% of the time or where it is not possible to isolate the effect of Tai Chi. - Aquatic exercises other than Tai Chi (e.g., aqua-aerobics, general resistance-based hydrotherapy, swimming, Pilates/yoga in water) or qigong in water without explicit classification as Tai Chi/Ai Chi. - Interventions shorter than 6 weeks, single sessions, or lack of data on dosage.
C	- Aquatic exercises not involving Tai Chi, if dose/format is reported. - Land-based Tai Chi (comparison of aquatic vs. land-based mode). - Standard land-based balance training. - Low-intensity activities: stretching, walking. - No intervention / usual care.	- Multicomponent high-intensity programs not matched to the intervention, preventing attribution of the effect. - Comparators including Tai Chi performed in water (identical intervention).
O	- Validated indicators of balance (static and/or dynamic) - Time points: post-intervention and/or follow-up (if available).	- Lack of at least one validated balance-related outcome. - Exclusively self-reported feelings of stability without the use of a validated tool
S	- Randomized and non-randomized controlled trials	- Case reports, meta-analyses, reviews, cross-sectional studies

### 2.2 Search strategy

Two authors (G.M. and K.K.) conducted a search in July 2025 in the following data-bases: PubMed, Scopus, EBSCO, and Web of Science, in order to identify thematically related publications. Extensive search strings tailored to the specificity of each scientific database were used. No time restrictions were set for the publication date. Articles in English were searched for, and no additional filters were applied. Detailed search commands for each database are presented below:

#### 2.2.1 Scopus

TITLE-ABS-KEY ((“tai chi” OR “tai ji” OR “tai ji quan” OR “tai chi chuan” OR “tai ji chuan” OR taiji OR taijiquan OR “t&apos;ai chi” OR “ai chi” OR qigong) AND (aquatic* OR “water-based” OR “water based” OR hydrotherap* OR “aquatic therap*” OR “aquatic exercis*” OR “water exercis*” OR hydrokinesiotherap* OR aquatherap* OR pool*) AND (balanc* OR “postural stabil*” OR “postural control” OR “postural balanc*” OR equilibrium OR “center of pressure” OR “centre of pressure” OR COP OR posturograph* OR sway OR “timed up and go” OR TUG OR “berg balance scale” OR “mini-bestest” OR “star excursion balance test” OR “functional reach” OR “y balance”) AND (elder* OR “older adult*” OR ageing OR aging OR geriatric* OR senior* OR “community-dwelling” OR “community dwelling”)).

#### 2.2.2 Web of science

TS = ((“tai chi” OR “tai ji” OR “tai ji quan” OR “tai chi chuan” OR “tai ji chuan” OR taiji OR taijiquan OR “t’ai chi” OR “ai chi” OR qigong) AND (aquatic* OR “water-based” OR “water based” OR hydrotherap* OR “aquatic therap*” OR “aquatic exercis*” OR “water exercis*” OR hydrokinesiotherap* OR aquatherap* OR pool*) AND (balanc* OR “postural stabil*” OR “postural control” OR “postural balanc*” OR equilibrium OR “center of pressure” OR “centre of pressure” OR COP OR posturograph* OR sway OR “timed up and go” OR TUG OR “berg balance scale” OR “mini-bestest” OR “star excursion balance test” OR “functional reach” OR “y balance”) AND (elder* OR “older adult*” OR ageing OR aging OR geriatric* OR senior* OR “community-dwelling” OR “community dwelling”)).

#### 2.2.3 PubMed

(“tai chi”[tiab] OR “tai ji”[tiab] OR “tai ji quan”[tiab] OR “tai chi chuan”[tiab] OR “tai ji chuan”[tiab] OR taiji[tiab] OR taijiquan[tiab] OR “t’ai chi”[tiab] OR “ai chi”[tiab] OR qigong[tiab]) AND (aquatic*[tiab] OR “water-based”[tiab] OR “water based”[tiab] OR hy-drotherap*[tiab] OR “aquatic therap*”[tiab] OR “aquatic exercis*”[tiab] OR “water exercis*”[tiab] OR hydrokinesiotherap*[tiab] OR aquatherap*[tiab] OR pool*[tiab]) AND (balanc*[tiab] OR “postural stabil*”[tiab] OR “postural control”[tiab] OR “postural balanc*”[tiab] OR equilibrium[tiab] OR “center of pressure”[tiab] OR “centre of pressure”[tiab] OR COP[tiab] OR posturograph*[tiab] OR sway[tiab] OR “timed up and go”[tiab] OR TUG[tiab] OR “berg balance scale”[tiab] OR “mini-bestest”[tiab] OR “star excursion balance test”[tiab] OR “functional reach”[tiab] OR “y balance”[tiab]) AND (elder*[tiab] OR “older adult*”[tiab] OR ageing[tiab] OR aging[tiab] OR geriatric*[tiab] OR senior*[tiab] OR “community-dwelling”[tiab] OR “community dwelling”[tiab]) NOT (animals[tiab]).

#### 2.2.4 EBSCO

(MH “Tai Chi” OR TI(“tai chi” OR “tai ji” OR “tai ji quan” OR “tai chi chuan” OR “tai ji chuan”) OR AB(“tai chi” OR taiji OR taijiquan OR “t’ai chi” OR “ai chi” OR qigong)) AND (TI(aquatic* OR “water-based” OR hydrotherap* OR “aquatic therap*” OR “aquatic exercis*”) OR AB(aquatic* OR “water based” OR hydrotherap* OR “water exercis*” OR hydrokinesiotherap* OR aquatherap*) OR SU(aquatic exercise OR hydrotherapy)) AND (TI(balanc* OR “postural control” OR “postural stabil*” OR equilibrium OR “center of pressure”) OR AB(balanc* OR COP OR posturograph* OR sway OR “timed up and go” OR TUG OR “berg balance scale” OR “mini-bestest” OR “functional reach” OR “y balance”) OR SU(balance)) AND (TI(elder* OR “older adult*” OR ageing OR aging OR geriatric* OR senior*) OR AB(elder* OR “community dwelling” OR “community-dwelling”) OR SU(aged OR geriatric patients)).

### 2.3 Selection process

A random draw was conducted among the authors to select two individuals who would carry out the article selection process. Two persons (J.J-N and M.O.) were appointed and subsequently instructed by the remaining authors regarding the adopted inclusion and exclusion criteria. Duplicates were initially removed manually, and on the basis of titles and abstracts, articles that preliminarily met the criteria were identified. After this process, the authors retrieved the full texts of the articles. No cases were observed in which it was justified to contact the publication authors to obtain access to the full text. Subsequently, one of the authors (Ł.R.) performed the final selection based on the full texts in order to qualify them for inclusion in the review process.

### 2.4 Data collection process

Two co-authors (K.K. and G.M.) manually extracted the data without using any specialized data extraction tools. Previously, two authors (J.J-N. and M.O.) had removed the titles and information related to the publication authors from the articles selected for the extraction process, in order to ensure absence of bias. After independent review, both authors met to verify consensus on the data extracted from the studies. At this stage, another author (T.A.) checked the accuracy and consistency of the extracted data. Data were extracted from each publication that met the adopted PICOs criteria. Any discrepancies be-tween the authors were resolved through joint discussion, with the main author (G.M.) having the final decision.

#### 2.4.1 Data items

The data extraction process was carried out using a pre-designed preliminary form developed specifically for the purposes of this review. From each eligible study, the following categories of data were collected:

- Population characteristics: type of participants, sample size and basic demographic data.
- Intervention: details of the aquatic Tai Chi exercise program, including duration of the intervention, frequency and length of sessions, as well as any modifications or additional training components.
- Comparison group: description of the control conditions or alternative intervention.
- Study type: type of study design-randomized controlled trial or non-randomized study.
- Balance assessment tools: tests and scales used to measure balance, as well as information on whether static, dynamic or functional balance was assessed.
- Time points of measurements: time points at which outcomes were assessed—i.e. before the start of the intervention and after its completion; additionally, any further follow-up measurements were recorded, if performed in the study.
- Outcomes: quantitative data for indicators of static and dynamic balance. The main extracted variables were the results of functional tests such as the Berg Balance Scale (BBS), Timed Up and Go (TUG), Tinetti test (POMA) and Five Times Sit-to-Stand (FTSTS). In the absence of raw data (means/standard deviations), estimated effect sizes or percentage changes reported by the authors were extracted.

### 2.5 Risk of bias assessment

Two validated research tools in the form of questionnaires were used to assess the risk of systematic error. The assessment was carried out independently by two authors (M.O. and Ł.R.). Any discrepancies were resolved through discussion and final approval by a third author (T.A.). For randomized controlled trials (RCTs), the ROB 2.0 (Risk of Bias 2.0) tool was applied, taking into account, among others, the randomization process, deviations from intended interventions, missing data, outcome measurement and selection of the reported results. For non-randomized studies, the ROBINS-I (Risk Of Bias In Non-randomized Studies of Interventions) tool was used, which assesses risk of bias in domains such as confounding factors, selection of participants into the study, classification of interventions, deviations from intended interventions, missing outcome data, out-come measurement and selective reporting.

### 2.6 Effect measures

The primary effect measure for continuous variables was the mean difference with a 95% confidence interval when the studies used the same measurement scale. In cases where identical outcomes were measured with different tools, the use of the standardized mean difference was considered. If the original studies reported *p*-values for between-group differences or within-group changes, these were recorded and included in the description of the results. For studies reporting only medians and interquartile ranges or results from statistical models, the estimated magnitudes of change or within- and between-group effect sizes were extracted and presented in accordance with the authors’ reporting.

### 2.7 Synthesis methods

Due to the anticipated clinical heterogeneity, meaning different populations (Parkinson’s disease, intellectual disability, healthy individuals) as well as methodological heterogeneity, a narrative synthesis supported by tabular summaries was performed, in accordance with the SWiM (Synthesis Without Meta-analysis) guidelines (24). The studies were grouped according to the type of control/comparator group to facilitate interpretation of the results:

- Ai Chi vs. land-based exercise.
- Ai Chi vs. other forms of aquatic therapy.
- Ai Chi vs. cognitive interventions/no activity.

The analysis included an assessment of the consistency of findings across studies, the strength of evidence for individual balance indicators, and a comparison of between group effect sizes where possible. The results were presented in a summary table including the characteristics of the study population, details of the intervention, and precise quantitative data for key balance indicators.

## 3 Results

### 3.1 Study selection

**Figure 1:**
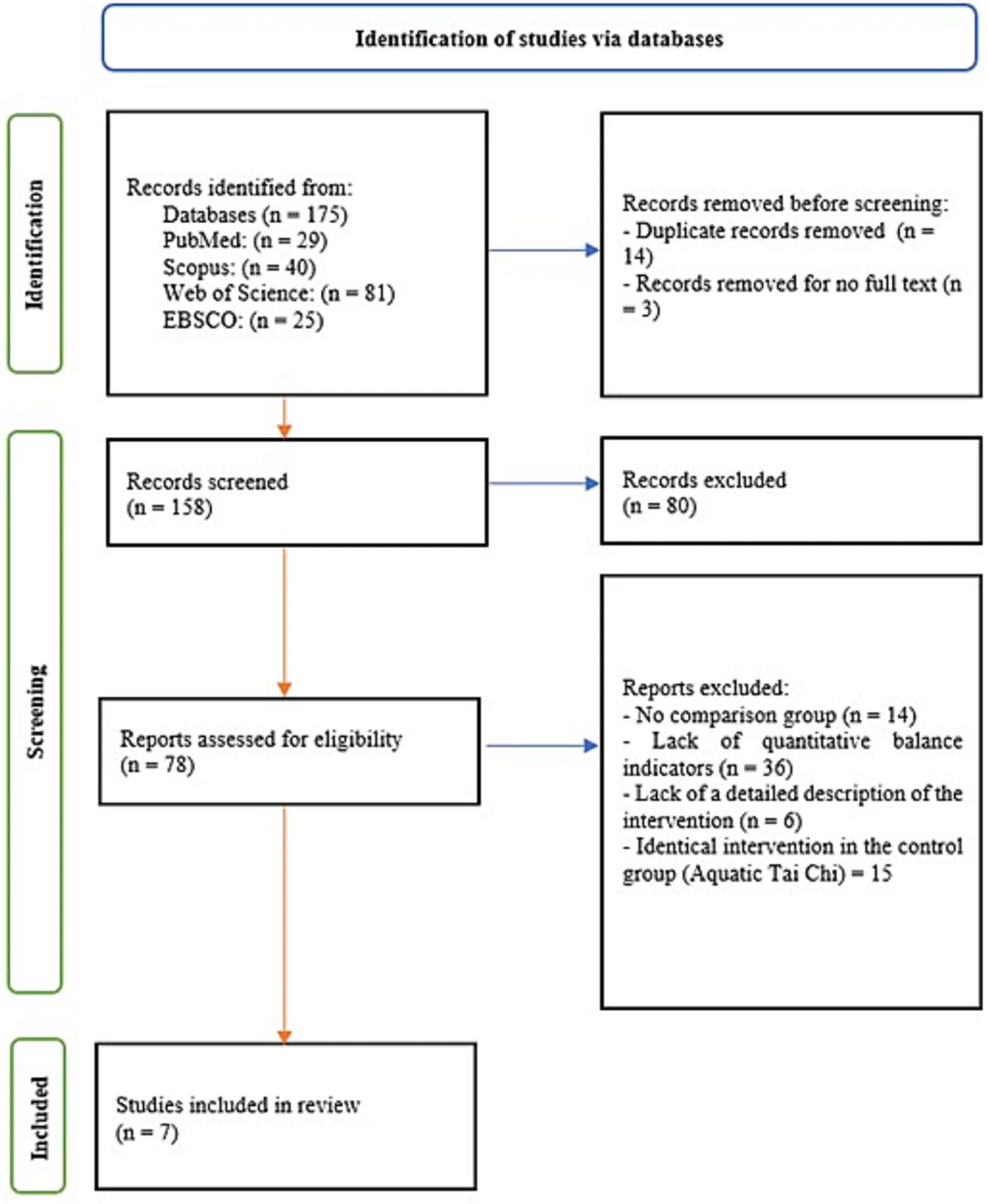
PRISMA flowchart (23).

A total of 175 records were identified in the search results from the four databases (PubMed, Scopus, Web of Science, EBSCO) without date restrictions. After removing 14 duplicates and three papers without access to the full text, 158 records remained for screening. Of these, 80 were excluded after title and abstract assessment as unrelated to the topic or not meeting the inclusion criteria. The full texts of 78 publications were assessed against the PICOs criteria; ultimately, 7 studies met the inclusion criteria and were included in the review. The most common reasons for exclusion at the full-text stage were: lack of a control group, absence of objective quantitative balance indicators, lack of a detailed description of the intervention, or inclusion of an identical intervention in the control group. All 7 studies meeting the criteria were included in the analysis, together with the characteristics of their populations, interventions and key outcomes (Figure 1).

### 3.2 Risk of bias analysis

**Figure 2:**
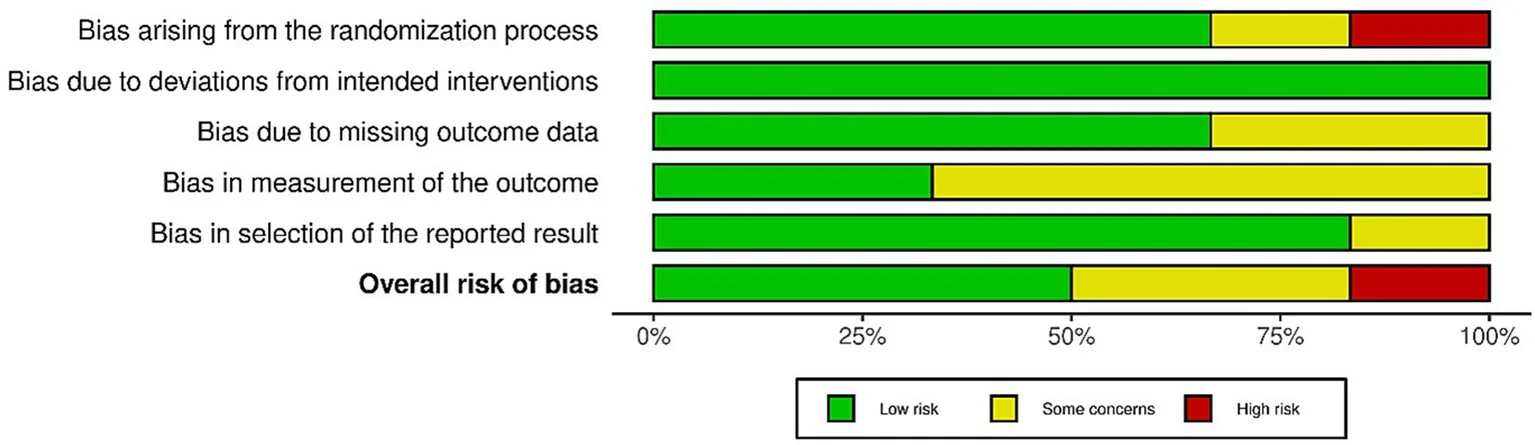
ROB-2 assessment of RCT.

**Figure 3:**
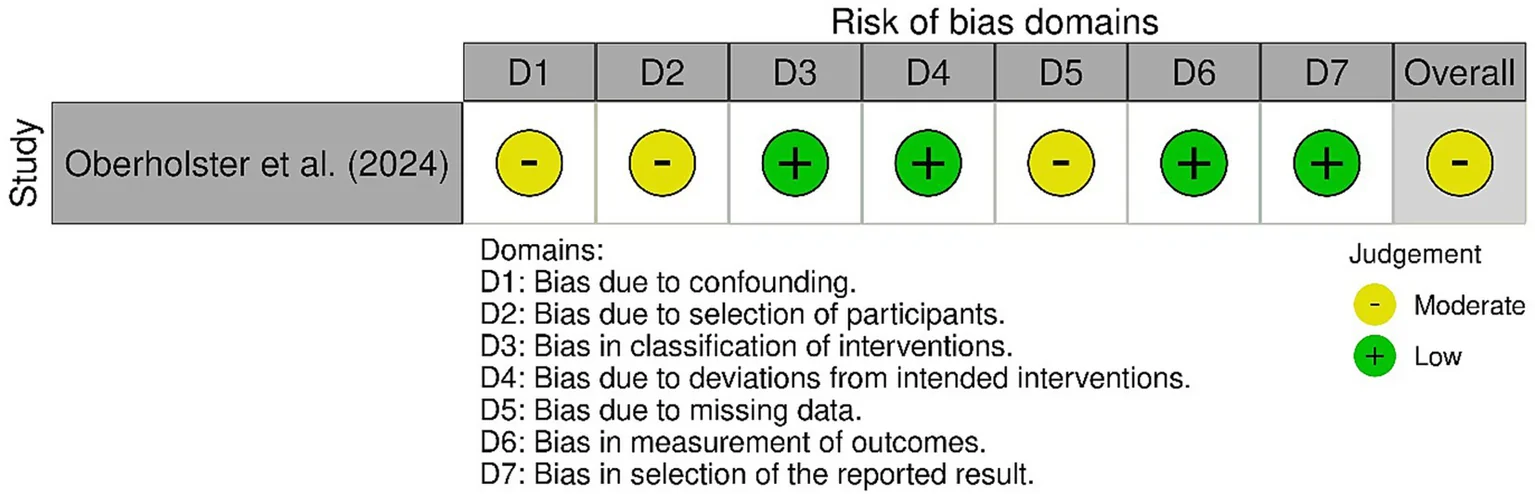
ROBINS-I assessment of non-randomized studies.

**Figure 4:**
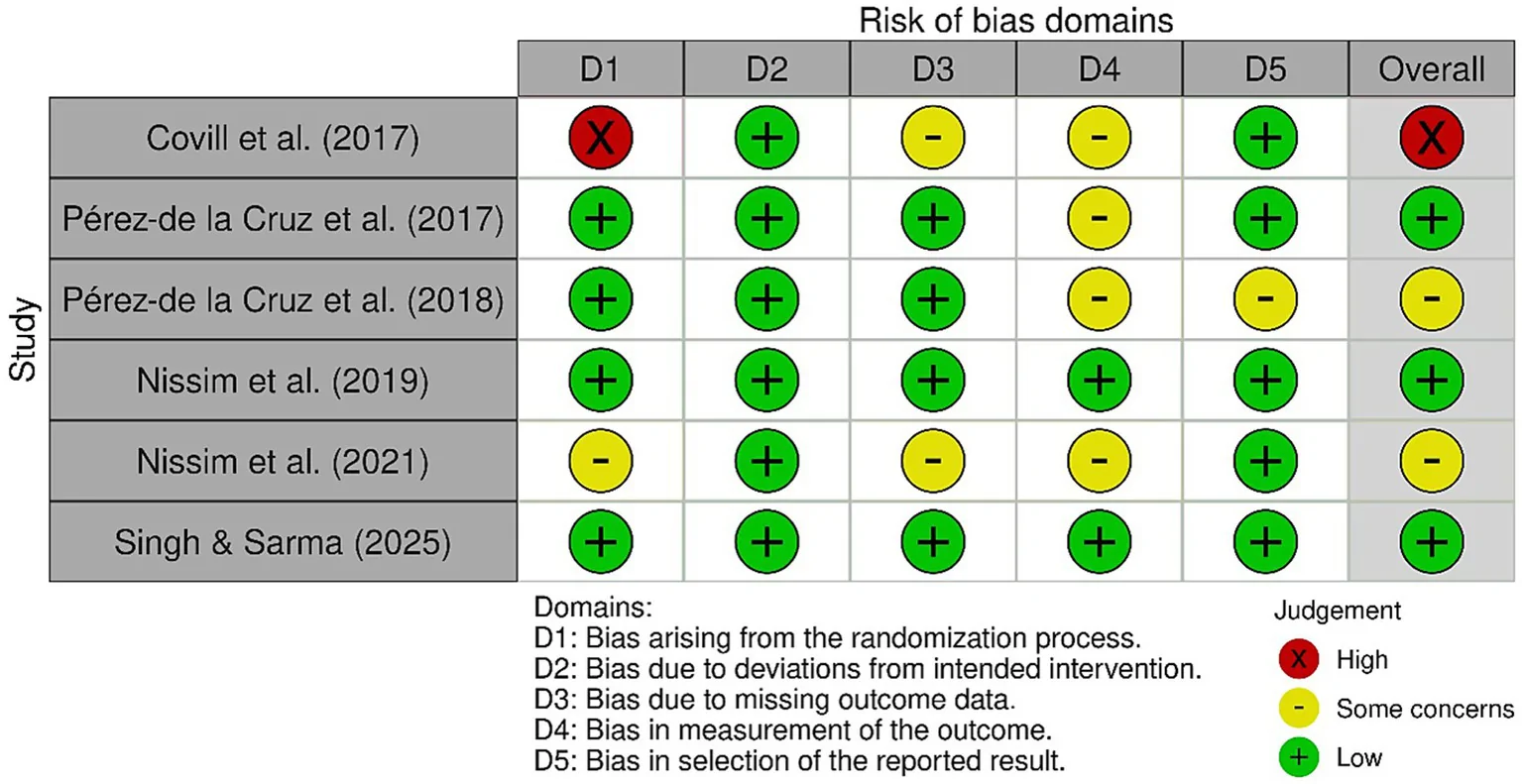
ROB-2 assessment of RCT.

Detailed results are presented in Figures 2, 3. The randomized controlled trials assessed using the ROB-2 tool were classified as follows: one study with a high risk of bias (12), three with a low risk of bias (4, 19, 21), and two as having “some concerns” (25, 26). In the case of the only CCT study, where the ROBINS-I tool was applied, it was classified as having a moderate risk of bias (20) (see Figure 4).

### 3.3 Study characteristics

A detailed description of the included studies is presented in Table 2. The total number of participants was *n* = 213, with mean ages ranging from 58 to 75 years (4, 12, 19–21, 25, 26). Sample sizes in individual studies ranged from 19 to 41 participants. Five studies were randomized controlled trials (4, 12, 19, 21, 26), one was defined as a controlled pilot RCT (small scale) (25), and one was a controlled clinical trial without full randomization (20). The studies were conducted in India, Spain, Israel, the USA and Australia. The study population included older adults with different clinical profiles: Parkinson’s disease (*n* = 2) (19, 25), intellectual disability (*n* = 1) (21), general balance deficits or fall risk (*n* = 3) (4, 12, 20), and older adults without severe diseases (*n* = 1) (26). In all studies, the experimental intervention was a structured aquatic exercise program in the form of Aqua Tai Chi. Ai Chi (Aquatic Tai Chi) interventions lasted from 6 to 14 weeks, with a frequency of 1 to 3 sessions per week. Control groups included: land-based therapy (balance exercises, Tai Chi), other forms of aquatic therapy (Halliwick, IBAT) or cognitive interventions (motor imagery).

**Table 2:** Characteristics of the studies and main outcomes for balance parameters.

Study	Participants	Study design	Ai Chi intervention (dose)	Control/Comparator group	Balance tests	Quantitative outcomes (mean ± SD or CI) (pre → post/change)	Main findings
Covill et al. (12)	N = 32 Age: 72.2 (I) vs. 75.1 (C) Balance deficits	RCT	6 weeks, 2×/week, 40-min sessions	Impairment-Based Aquatic Therapy (aquatic therapy focused on balance deficits)	Berg Balance Scale (BBS), Timed Up & Go (TUG)	BBS (I): 45.1 ± 6.3 → 47.6 ± 4.4 BBS (C): 42.1 ± 10.4 → 46.3 ± 5.6 TUG (I): 16.1 ± 6.0 → 14.2 ± 4.9 s TUG (C): 16.9 ± 3.8 → 15.8 ± 5.1 s	No significant between-group differences for BBS (*p* = 0.53) and TUG (*p* = 0.39). Both aquatic interventions produced within-group improvement, but Ai Chi was not more effective than the specific aquatic training.
Pérez-de la Cruz (19)	N = 30 Age: 66.8 (I) vs. 67.5 (C) Parkinson’s disease (H&Y 2–3)	RCT	10 weeks, 2×/week, 60-min sessions	Land-based exercises (standard rehabilitative physiotherapy)	BBS, Tinetti (POMA), 5 x Sit-to-Stand (FTSTS), Timed Up & Go	Tinetti (I): 19.1 ± 6.2 → 21.7 ± 5.8 Tinetti (C): 19.3 ± 6.6 → 19.0 ± 6.4 BBS (I): 40.0 ± 9.6 → 44.1 ± 7.5 BBS (C): 39.4 ± 8.8 → 39.4 ± 8.8 TUG (I): 11.6 → 9.1	Ai Chi showed superiority over the control group (time × group interaction) for Tinetti (*F* = 20.1, *p* < 0.001), BBS (*F* = 19.6, *p* < 0.001) and TUG (*F* = 11.7, *p* < 0.001). No clinically relevant between-group difference for FTSTS (*p* = 0.006, but with a smaller effect).
Pérez-de la Cruz (25)	N = 29 Age: 65.9 (I) vs. 66.4 (C) Parkinson’s disease (H&Y 1–3)	Controlled pilot study (RCT)	11 weeks, 2×/week, 45-min sessions	Conventional land-based exercises (general rehabilitative physiotherapy)	TUG, 5 × STS, single-leg stance (time maintaining balance on each leg)	TUG (I): 11.1 ± 6.4 → 8.8 ± 2.4 (Follow-up) TUG (C): 11.5 ± 2.2 → 11.4 ± 2.7 FTSTS (I): 17.8 ± 5.5 → 14.3 ± 3.2 (Follow-up) FTSTS (C): 18.3 ± 4.8 → 18.0 ± 4.1	A significant group × time interaction in favour of Ai Chi for TUG (*F* = 11.6, *p* < 0.001) and SLS (*F* = 29.8, *p* < 0.001).
Nissim et al. (21)	N = 41 Age: 58.2 (I) vs. 58.9 (C) Intellectual disability (ID)	RCT	14 weeks, 1×/week, 30–45-min sessions	Land-based Tai Chi (identical sequence as in water)	Tinetti Balance & Gait (TBAT—total score for balance and gait, 0–28 points)	Tinetti Total (I): 25.3 ± 2.5 → 26.3 ± 2.1 Tinetti Total (C): 24.9 ± 2.3 → 25.8 ± 2.2 Balance (I): 14.4 ± 1.9 → 14.8 ± 1.5 Balance (C): 14.2 ± 1.7 → 14.6 ± 1.7	Both groups improved their Tinetti scores. The Ai Chi group showed faster improvement (significant differences in changes between T1–T2 and T2–T3 in favour of Ai Chi in the early phase). No significant difference in the final outcome between the groups.
Nissim et al. (26)	N = 19 Age: 73.3 (I) vs. 74.0 (C1) vs. 77.0 (C2) Healthy older adults	Controlled pilot study (RCT)	12 weeks, 2×/week, 30-min sessions	Land-based Ai Chi and guided imagery	Tinetti balance sub-test (POMA, balance component 0–16 points)	Tinetti (I): 13.5 ± 1.8 → 15.0 ± 1.5 Tinetti (C1): 13.8 ± 1.4 → 14.4 ± 1.6 Tinetti (C2): 14.5 ± 0.5 → 14.6 ± 0.5	A significant time × group interaction (*F* = 3.74, *p* < 0.05). Only the Ai Chi (aquatic) group showed a significant within-group improvement (t = −4.8, *p* < 0.01). The Ai Chi group achieved greater improvement than both control groups.
Oberholster et al. (20)	N = 32 Age: 68.6 (I) vs. 71.5 (C1) vs. 78.5 (C2) Fall risk/neurological conditions	CCT	8 weeks, 2×/week, 45–50-min sessions	Land-based balance training program: high-level (dynamic balance exercises) or low-level (gentler functional training)	TUG, 5 × STS, Functional Reach Test (FRT), 6-min walk, 6-meter walk (time and number of steps), self-reported fall scales (including MFES)	TUG (I): Median 15.4 → Change: ↓ 10.4% 5 × STS (I): Median 19.2 → Change: ↓ 1.67 s 6MWT (I): Mean 280 m → Change: ↑ 9.43 m *(Time effect estimates from the mixed model)*	No differences in the intervention effect (no program × time interaction) were found between Ai Chi and the land-based programs (HLB/LLB). All programs produced similar improvements.
Singh and Sarma (4)	N = 30 Age: 71.6 (I) vs. 72.4 (C) Older adults with balance disorders	RCT	8 weeks, 3×/week, 40-min sessions	Balance–strength training in water without Tai Chi elements	BBS, TUG, Dynamic Gait Index (DGI)—measurements at baseline, after 4 weeks and after 8 weeks of the intervention	BBS (I): 39.9 ± 3.4 → 48.5 ± 3.2 BBS (C): 38.2 ± 5.1 → 43.9 ± 5.6 TUG (I): 12.8 ± 1.7 → 9.7 ± 2.2 s TUG (C): 13.9 ± 2.8 → 11.7 ± 2.7 s	The Ai Chi group achieved greater improvement than the control group. The mean difference (MD) for BBS between the groups after the intervention was 4.6 points in favour of Ai Chi (p < 0.001).

RCT, Randomized Controlled Trial; CCT, Controlled Clinical Trial (not fully randomized); BBS, Berg Balance Scale; TUG, Timed Up and Go Test; Tinetti/TBAT, Tinetti Balance Assessment Tool; FTSTS/5 × STS, Five Times Sit-to-Stand Test; DGI, Dynamic Gait Index; SLS, Single Leg Stance; 6MWT, 6-Minute Walk Test; ID, Intellectual Disability; I, Intervention Group; C, Control Group.

The control groups differed between studies, which reflects the lack of an established standard in this narrow research area. In four RCTs, the control group performed land-based training: in two studies these were conventional physiotherapy exercises focused on improving balance and function in patients with Parkinson’s disease, whereas in one pilot study involving healthy older adults, Ai Chi in water was compared with analogous Tai Chi exercises performed on land and with a control condition in the form of relaxation–imagery sessions. In three studies, Ai Chi was compared with other active aquatic interventions. Final outcomes in all studies included validated tests of dynamic and static balance.

### 3.4 Measures of effect

All balance outcomes were presented as changes in mean values of balance tests together with standard deviations or other measures of dispersion. Table 2 shows the means (± SD) in the Ai Chi and control groups before and after the intervention, which made it possible to compare the magnitude of improvement. In the narrative description of the results, between-group comparisons based on mean differences were used. Since individual studies employed different balance scales, the use of the standardized mean difference was considered in the planned quantitative synthesis in order to express the effect size of Ai Chi in a manner comparable across different tests. Ultimately, due to the heterogeneity of measurement methods and control interventions, a pooled meta-analysis was not performed, and the results were synthesized qualitatively, with 95% confidence intervals (CI) and *p*-values reported for key between-group differences, in accordance with the data presented in the original studies. A significance level of *p* < 0.05 was adopted. Where data from analyses of variance were available, the corresponding *F* values and effect sizes were also reported.

### 3.5 Syntheses of results: effectiveness on balance indicators

The effectiveness of the Ai Chi intervention was assessed primarily using functional tests: the Berg Balance Scale (BBS), Timed Up and Go (TUG), and the Tinetti Scale (POMA).

- Dynamic balance and mobility (TUG Test)—The TUG test was the most frequently used outcome measure; it was employed in 5 out of the 7 included studies (4, 12, 19, 20, 25). The results indicate heterogeneous effectiveness depending on the comparator group. In studies involving individuals with Parkinson’s disease (19, 25) and older adults in India (4), the Ai Chi intervention produced a statistically significantly greater reduction in TUG time compared with the control groups. In contrast, in the studies by Oberholster et al. (20) and Covill et al. (12), despite observed within-group improvements, no superiority of Ai Chi over specific therapeutic programs was demonstrated.
- Static and functional balance (Berg Balance Scale-BBS)—Three studies used the BBS. Singh and Sarma (4) and Pérez-de la Cruz (19) demonstrated a significant advantage of Ai Chi over the control groups, with mean differences suggesting clinically meaningful change. Covill et al. did not observe significant between-group differences, which suggests that Ai Chi may be equivalent to other forms of targeted aquatic therapy (12).
- Gait and balance assessment (Tinetti Scale and DGI)—In studies using the Tinetti scale, a positive effect of Ai Chi was observed (19, 21, 26). In a pilot study on older adults without severe diseases (26), only the Ai Chi group showed improvement. In the population with intellectual disability (21), Ai Chi produced faster effects than land-based Tai Chi, although the final outcome was similar. For the Dynamic Gait Index (DGI), Singh & Sarma showed superiority of Ai Chi over Halliwick therapy (4).

## 4 Discussion

The Available clinical studies indicate that Ai Chi therapy is associated with potential improvement in balance and mobility parameters in older adults and neurological patients. Covill et al. (12) showed that both Ai Chi and conventional aquatic therapy improved Berg Balance Scale (BBS) and Timed Up and Go (TUG) scores in older adults with balance disorders. Pérez-de la Cruz reported that an Ai Chi program significantly reduced perceived pain and improved functional status in patients with Parkinson’s disease, out-performing land-based therapy (19). Similarly, Pérez-de la Cruz found that Ai Chi contributed to a reduction of motor symptoms, particularly bradykinesia and rigidity, and improved motor outcomes in people with Parkinson’s disease (25). Nissim et al. compared Ai Chi and Tai Chi in older adults with intellectual disability-both interventions reduced fall risk, as reflected by improvements in the Tinetti test, with the aquatic group achieving faster improvement and additionally showing enhanced verbal memory (21). In turn, Nissim and Livny demonstrated that regular aquatic exercise may be effective in improving balance in older adults compared to Tai Chi, an intervention performed on land. Additionally, according to the study findings, Ai Chi reduced activation in the left cerebellar hemisphere. According to other studies, this is considered a positive aspect, as the contribution of the cerebellar cortex should decrease with increasing task proficiency (26, 27). In the study by Oberholster et al. (20), Ai Chi, as part of a fall-prevention program, led to lasting improvement in functional parameters, with gains maintained for 6 months, comparable to land-based programs. More recent work points to the superiority of Ai Chi: Singh and Sarma (4) showed that in older patients from India, Ai Chi significantly increased BBS scores, shortened TUG times and improved Dynamic Gait Index results to a greater extent than standard aquatic exercises. Taken together, Ai Chi therapy was associated with improvements in static and dynamic balance, mobility and pain reduction, and in many studies produced outcomes equivalent or superior to comparator interventions such as land-based therapy and other forms of hydrotherapy (4, 19).

The analysed studies included diverse groups of patients at risk of balance disorders. Covill et al. examined 32 healthy individuals aged 65–85 years with balance impairments receiving pool-based therapy (12). Pérez-de la Cruz (19, 25) focused on patients with idiopathic Parkinson’s disease in Hoehn–Yahr stages 1–3, aged approximately 40–80 years. Nissim et al. (21) studied 41 individuals aged 50–66 years with mild or moderate intellectual disability. Nissim et al. (26) included 19 adults aged 65 to 85 years in the study, all of whom scored below 10 points on the Geriatric Depression Scale. Singh and Sarma (4) included 30 independently living seniors in India aged 65–80 years with diagnosed balance deficits. The clinical relevance of these studies is underscored by the eligibility criteria: Parkinson’s patients were assessed in the “off” state, with normal MMSE scores, without advanced dementia or medical contraindications, which makes the findings applicable to this population (19, 28, 29). Balance impairment in the studied groups often corresponds to increased fall risk, activity limitation and reduced quality of life (30). Therefore, demonstrating the effectiveness of Ai Chi in such populations, particularly where it exceeds or matches land-based therapy, has important rehabilitative implications and highlights the value of this program as a safe, low-impact tool for clinicians (31).

Ai Chi is based on slow, coordinated whole-body movements combined with deep breathing, aimed at muscle relaxation, postural strengthening and balance training (25, 32). The aquatic environment supports these effects through specific physical properties of water, such as buoyancy, density, hydrostatic pressure and turbulence, which simultaneously enable increased body stabilization and resistance (20, 33). Water buoyancy unloads the joints of the lower limbs, reducing fear of falling, while viscous resistance and movement speed facilitate safe coordination training (34–36). Hydrostatic pressure improves sensory perception of the body (proprioception) and supports blood flow and respiratory function (37). Water resistance requires motor control with each movement, which is important because many Ai Chi exercises involve trunk rotation, limb elevation and changes in stance width coordinated with breathing (12, 20). These conditions may stimulate adaptive neurodevelopmental mechanisms, for example by improving the ability to maintain balance under altered sensory inputs (37). In addition, mindfulness elements related to slow breathing may reduce muscle tension and perceived pain (38–40). Although the precise physiological pathways are not fully elucidated, reports suggest increased cerebellar activation and changes in sensory integration following Ai Chi training, which may translate into better postural stabilization (20).

Most studies used well-established clinical tests for the assessment of balance and mobility. The most frequently used tools were the Berg Balance Scale (BBS)-a postural balance scale used by Covill et al. (12) and Singh and Sarma (4), which assesses static and partly dynamic balance—and the Timed Up and Go (TUG) test, measuring the time to stand up from a chair, walk 3 m and return, employed for example by Covill et al. (12), Pérez-de la Cruz (19), and Singh and Sarma (4); it is a simple indicator of functional mobility. In the studies by Pérez-de la Cruz (19) and Nissim (21), the Tinetti test (Tinetti Balance and Gait Test, TBAT) was used, which assesses both balance and gait. Singh and Sarma (4) added the Dynamic Gait Index (DGI), which measures stability while walking under path variations and is often used to predict falls (41). Other tools included the Five Times Sit-to-Stand test, assessing lower-limb strength, single-leg stance tests evaluating static stability, and questionnaires such as the Activities-specific Balance Confidence scale (in Covill) or PDQ-39 (Parkinson’s Disease Questionnaire-39) for quality of life in patients with Parkinson’s disease. It is worth noting that these tools differ in sensitivity and scope. For example, changes in BBS must reach several points to be clinically meaningful, whereas TUG is sensitive to overall functional capacity. The combined results of several tests make it possible to better capture the effects of aquatic therapy on various aspects of balance-static and dynamic, conscious and automatic postural components—but require consideration of their limitations, including ceiling or floor effects.

Ai Chi also affects pain symptoms and psychosocial aspects. Pérez-de la Cruz showed that Ai Chi therapy significantly reduced pain as measured by the VAS scale in patients with Parkinson’s disease (19). The effect was likely related to muscle relaxation and enhanced blood flow. Covill et al. (12), on the other hand, found no significant changes in self-reported pain (NPRS) after either aquatic program (12), which may reflect differences in populations or protocols. With regard to quality of life, Pérez-de la Cruz (19) observed improvements in PDQ-39 subscales in Parkinson’s patients after Ai Chi. Pérez-de la Cruz (25) reported no significant changes in overall PDQ-39, except for higher perceived social support in the Ai Chi group, suggesting that participation in a water-based group may enhance a sense of belonging and motivation. Additionally, the slow, relaxing components of Ai Chi may strengthen balance self-confidence and self-efficacy, although most studies did not assess these variables directly. It is possible that Ai Chi brings not only motor benefits but also alleviates chronic pain and improves patients’ subjective experiences such as quality of life and emotional support, making this therapy a comprehensive rehabilitation tool. The obtained results should also be analyzed in the context of contemporary literature on fall prevention. Recent meta-analyses indicate that classical land-based Tai Chi effectively improves dynamic balance and motor function, which directly translates into a reduced risk of falls among older adults (42). Nevertheless, for a substantial proportion of the geriatric population, traditional exercise programs may constitute a barrier due to excessive loading of the musculoskeletal system. An alternative to these interventions is the use of the aquatic environment in the therapy of such patients. Melo et al. (43) report that targeted aquatic physiotherapy significantly improves gait parameters and overall quality of life in older adults, while effectively reducing fear of falling. The conclusions drawn from the present analysis are also fully consistent with the most recent findings of Singh and Sarma. In their own research context, the authors demonstrated that Ai Chi interventions significantly reduced time in the TUG test and improved scores on the BBS scale compared with conventional hydrotherapy (4).

The review reveals considerable heterogeneity of results and methodological limitations. The demonstrated effects of Ai Chi are sometimes inconsistent: for example, Covill found no differences between Ai Chi and another aquatic exercise program, whereas Singh reported a clear advantage of Ai Chi over another aquatic therapy (4, 12). Some in-consistencies arise from differences in populations regarding age or comorbidities, program durations ranging from 6 to 14 weeks, and training intensity. Training loads, exercise selection and number of sessions varied widely. In addition, many studies had small sample sizes and design shortcomings affecting study quality: Covill et al. is a small-sample study without blinding, Nissim et al. describes a pilot trial with 41 participants, and the study by Oberholster et al. was an uncontrolled clinical trial with a high dropout rate of 43% (12, 20, 21). Many outcomes relied on self-report or short-term measurements without long-term follow-up, which limits conclusions about the durability of benefits. Differences in the tools used make it difficult to compare effectiveness across studies. The authors themselves point to the need for protocol standardisation and more reliable measurements. For instance, Covill stresses the necessity of further research to optimise training frequency and duration, while Oberholster highlights potential selection bias and the need for randomisation of participants and full registration of clinical programs (12, 20). Consequently, although most findings are promising, caution is required when generalising, and larger, better-controlled clinical trials are needed.

Future studies should focus on determining the optimal parameters of Ai Chi therapy, such as the duration and frequency of sessions, and on confirming the observed effects in larger, multicentre randomized trials. Long-term follow-up after completion of the intervention is also essential to evaluate the durability of benefits. It will be important to conduct studies comparing Ai Chi with other forms of neurological rehabilitation, such as PNF-based methods (Proprioceptive Neuromuscular Facilitation), as well as those combining aquatic training with cognitive–motor therapy (44, 45). The potential of Ai Chi in patients with multiple sclerosis or stroke should be explored more extensively, as current data suggest effectiveness in this group. It is also necessary to evaluate the neural mechanisms using various imaging modalities or EMG measurements, which may clarify how Ai Chi modifies muscle activation or cerebellar activity (46, 47). Additionally, the impact of Ai Chi on psychosocial aspects such as fear of falling and group interactions in the local community should be investigated to objectify benefits observed in subjective questionnaires (48–50). Ultimately, implementation research on integrating Ai Chi into routine practice will help assess its economic and social impact within the healthcare system.

Ai Chi therapy appears to be a safe and effective method for complementing rehabilitation in older adults and neurological patients, especially when traditional land-based exercises are limited for any reason. Thanks to the supportive aquatic environment, Ai Chi exercises can be successfully applied in patients with motor limitations, leading to improved balance and reduced symptoms without overloading the joints. In the Parkinson’s disease population, Ai Chi has proved more effective than analogous land-based training in improving balance and quality of life. Clinical data suggest that short programs of ap-proximately 8 to 12 weeks lead to immediate improvements in motor parameters that can be maintained for several months. In therapeutic practice, it is worth considering the inclusion of Ai Chi in comprehensive rehabilitation programs, particularly in patients who are reluctant to engage in traditional exercise. However, due to study limitations, careful individualisation of therapy and monitoring of outcomes are advisable. Overall, there is rationale to recommend Ai Chi as a cost-effective and engaging method for improving balance, functioning and quality of life in older adults and individuals with neurological disorders.

### 4.1 Limitations of the study

For Despite the rigorous methodological process, this systematic review has certain limitations that should be considered when interpreting the results. First, the majority of the included studies were characterized by small sample sizes (ranging from *N* = 19 to *N* = 41). Small sample sizes limit the statistical power of tests and increase the risk of Type II error, and may also lead to an overestimation of the therapeutic effect size.

Furthermore, the methodological quality of the analyzed studies was heterogeneous. The review included both randomized controlled trials (RCTs) and non-randomized studies (CCT/quasi-experimental). In the case of RCTs, a frequent issue was the lack of blinding of participants and therapists, which is difficult to avoid in aquatic physical therapy research but may introduce performance bias. The study by Oberholster et al. did not employ randomization, which increases the risk of selection bias and confounding factors. Another limitation is the lack of long-term follow-up. Only a few studies assessed the sustainability of therapeutic effects after the intervention had ended. Consequently, it remains unclear whether the balance improvement achieved through Ai Chi persists in the long term without the continuation of exercises.

Finally, due to the heterogeneity of measurement tools and the diversity of control groups, it was not possible to conduct a meta-analysis. Therefore, the synthesis of results relies on narrative analysis, which, despite the diligence exercised, is more susceptible to subjective interpretation than the statistical pooling of data.

## 5 Conclusion

This systematic review provides evidence that Aquatic Tai Chi is an effective and safe therapeutic intervention that leads to improvements in static and dynamic balance in individuals over 60 years of age, both healthy and those with neurological conditions. The analysis showed that in many cases Ai Chi appears comparable or superior with respect to selected outcomes, particularly in improving functional mobility as measured by the TUG test and postural stability as assessed by the Berg Balance Scale. Compared with other forms of hydrotherapy, Ai Chi shows the potential to generate better outcomes than conventional group exercises, although when contrasted with specific task-oriented aquatic training, the effects may be comparable.

It is recommended that Ai Chi be incorporated into standard fall-prevention and geriatric rehabilitation programs as an alternative or complement to land-based therapy. However, further high-quality randomized controlled trials (RCTs) with long-term follow-up are necessary to precisely determine the optimal dosing parameters of the intervention, including the frequency and duration required to achieve lasting effects.

### 5.1 Practical implications

Ai Chi may represent a useful intervention for older adults and neurological patients for whom traditional land-based exercises may be too demanding or associated with an increased risk of injury. Based on the conducted analysis, the implementation of short, structured programs lasting 8 to 12 weeks may yield measurable and relatively rapid improvements in motor parameters, particularly in patients who demonstrate reluctance toward conventional forms of physical activity.

## Data Availability

The original contributions presented in the study are included in the article/supplementary material, further inquiries can be directed to the corresponding authors.
