# Correction: Effectiveness of aquatic Tai Chi on balance indicators: a systematic review

**DOI:** 10.3389/fpubh.2026.2011862

**Published:** 2026-10-26

**Authors:** Grzegorz Mańko, Tadeusz Ambrozy, Krzysztof Kasicki, Jarosław Jaszczur-Nowicki, Mariusz Ozimek, Piotr Czech, Paweł Gąsior, Łukasz Rydzik

**Affiliations:** 1 Department of Biomechanics and Kinesiology, Institute of Physiotherapy, Jagiellonian University Collegium Medicum, Kraków, Poland; 2 Małopolska Rehabilitation Hospital in Krzeszowice, Kraków, Poland; 3 Department of Sport Theory and Motor Skills, Institute of Sports Sciences, University of Physical Culture in Kraków, Kraków, Poland; 4 Department Physiotherapy, School of Public Health, Collegium Medicum, University of Warmia and Mazury, Olsztyn, Poland; 5 Faculty of Physical Culture and Safety Sciences, Academy of Applied Sciences in Nowy Sącz, Nowy Sącz, Poland; 6 Institute of Humanities and Tourism, Academy of Applied Sciences in Nowy Targ, Nowy Targ, Poland

**Keywords:** aging, aqua therapy, older adults, exercise, fall risk, well being

In the published article, there were errors in the reference list and in several in-text citation assignments. The corrections are listed below.

The reference for 2 (Horak, 2006) was erroneously written as Horak FB. Postural orientation and equilibrium: what do we need to know about neural control of balance to prevent falls? *Age Ageing*. (2006) 35:ii7–ii11. doi: 10.1093/ageing/afl081. It should be Horak FB. Postural orientation and equilibrium: what do we need to know about neural control of balance to prevent falls? *Age Ageing*. (2006) 35(Suppl 2):ii7–ii11. doi: 10.1093/ageing/afl077.

The reference for 4 (Singh and Sarma, 2025) was erroneously written as Singh P, Sarma PC. Long-term efficacy of aquatic therapy on balance and gait in older adults: a randomised clinical trial. *J Clin Diagn Res*. (2025) 19:YC01–5. doi: 10.7860/JCDR/2025/78107.20945. It should be Singh P, Sarma PC. Effectiveness of Ai Chi as aquatic intervention for balance impairments in community-dwelling older adults in India: a randomised controlled study. *J Clin Diagn Res*. (2025) 19:YC01–6. doi: 10.7860/JCDR/2025/74990.20569.

The reference for 7 (Koh, 1981) was erroneously written as Koh TC. Tai Chi Chuan. *Am J Chin Med*. (1981) 9:15–22. doi: 10.1142/s0192415x81000036. It should be Koh TC. Tai Chi Chuan. *Am J Chin Med*. (1981) 9:15–22. doi: 10.1142/S0192415X81000032.

The reference for 10 (Stathokostas and Jacob-Johnson, 2015) was erroneously written as Stathokostas L, Jacob-Johnson S. “The risks of exercise for older adults”. In: Copeland J, editor. *Physical Activity for Older Adults*. Cham: Springer (2015). p. 29–40. It should be Stathokostas L, Jones G. “Risks of exercise for older adults.” In: Sullivan GM, Pomidor AK, editors. *Exercise for Aging Adults: A Guide for Practitioners*. Cham: Springer (2015). p. 29–39. doi: 10.1007/978-3-319-16095-5_3.

The reference for 14 (Mehrpour et al., 2020) was erroneously written as Mehrpour Z, Bagheri S, Letafatkar A, Mehrabian H. The effect of a water-based training program on pain, range of motion and joint position sense in elite female swimmers with impingement syndrome. *Phys Treat*. (2020) 10:223–32. doi: 10.32598/ptj.10.4.455.1. It should be Mehrpour Z, Bagheri S, Letafatkar A, Mehrabian H. The effect of a water-based training program on pain, range of motion and joint position sense in elite female swimmers with impingement syndrome. *J Adv Sport Technol*. (2020) 4:72–81. Available online at: https://jast.uma.ac.ir/article_930.html.

The reference for 15 (Ma et al., 2021) was erroneously written as Ma T, Song X, Ma Y, Hu H, Bai H, Li Y. The effect of thermal mineral waters on pain relief, physical function and quality of life in patients with osteoarthritis: a systematic review and meta-analysis. *Medicine (Baltimore)*. (2021) 100:e24601. doi: 10.1097/MD.0000000000024601. It should be Ma T, Song X, Ma Y, Hu H, Bai H, Li Y, et al. The effect of thermal mineral waters on pain relief, physical function and quality of life in patients with osteoarthritis: a systematic review and meta-analysis. *Medicine (Baltimore)*. (2021) 100:e24488. doi: 10.1097/MD.0000000000024488.

The reference for 17 (Torres-Ronda and Shelling, 2014) was erroneously written as Torres-Ronda L, Shelling X. The properties of water and their applications for training. *J Hum Kinet*. (2014) 44:237–52. doi: 10.2478/hukin-2014-0129. It should be Torres-Ronda L, Schelling i del Alcázar X. The properties of water and their applications for training. *J Hum Kinet*. (2014) 44:237–48. doi: 10.2478/hukin-2014-0129.

The reference for 28 (Kurt et al., 2018) was erroneously written as Kurt EE, Büyükturan B, Büyükturan Ö, Erdem HR, Tuncay F. Effects of AI Chi on balance, quality of life, functional mobility, and motor impairment in patients with Parkinson's disease. *Disabil Rehabil*. (2018) 40:791–7. doi: 10.1080/09638288.2017.1312658. It should be Kurt EE, Büyükturan B, Büyükturan Ö, Erdem HR, Tuncay F. Effects of Ai Chi on balance, quality of life, functional mobility, and motor impairment in patients with Parkinson's disease. *Disabil Rehabil*. (2018) 40:791–7. doi: 10.1080/09638288.2016.1276972.

The reference for 29 (Morris, 2010) was erroneously written as Morris DM. Aquatic therapy to improve balance dysfunction in older adults. *Top Geriatr Rehabil*. (2010) 26:104–19. doi: 10.1097/TGR.0b013e3181e8538d. It should be Morris DM. Aquatic therapy to improve balance dysfunction in older adults. *Top Geriatr Rehabil*. (2010) 26:104–19. doi: 10.1097/TGR.0b013e3181dfda0a.

The reference for 31 (Seijas et al., 2023) was erroneously written as Seijas V, Kiekens C, Gutenbrunner C, Nugraha B. Advancing the world health assembly's landmark resolution on strengthening rehabilitation in health systems: unlocking the future of rehabilitation. *J Rehabil Med*. (2023) 55:jrm5413. doi: 10.2340/jrm.v55.5413. It should be Seijas V, Kiekens C, Gimigliano F. Advancing the World Health Assembly's landmark resolution on strengthening rehabilitation in health systems: unlocking the future of rehabilitation. *Eur J Phys Rehabil Med*. (2023) 59:447–51. doi: 10.23736/S1973-9087.23.08160-1.

The reference for 32 (Çaǧlar and Taşkıran, 2024) was erroneously written as Çaǧlar E. Aquatic exercise and potential health effects. *Qubahan Acad J*. (2024) 4:337–44. doi: 10.61707/jpdvkn71. It should be Çaǧlar EÇ, Taşkıran C. Aquatic exercise and potential health effects. *Int J Relig*. (2024) 5:337–44. doi: 10.61707/jpdvkn71.

The reference for 33 (Coraci et al., 2022) was erroneously written as Coraci D, Tognolo L, Maccarone M. Water-based rehabilitation in the elderly: data science approach to support the conduction of a scoping review. *Appl Sci*. (2022) 12:4529. doi: 10.3390/app12094529. It should be Coraci D, Tognolo L, Maccarone MC, Santilli G, Ronconi G, Masiero S. Water-based rehabilitation in the elderly: data science approach to support the conduction of a scoping review. *Appl Sci*. (2022) 12:8999. doi: 10.3390/app12188999.

The reference for 35 (Pugh et al., 2015) was erroneously written as Pugh CJA, Sprung VS, Ono K, Spence AL, Thijssen DHJ, Carter HH, et al. The effect of water immersion during exercise on cerebral blood flow. *Med Sci Sports Exerc*. (2015) 47:299–306. doi: 10.1249/MSS.000000000000042224977699. It should be Pugh CJA, Sprung VS, Ono K, Spence AL, Thijssen DHJ, Carter HH, et al. The effect of water immersion during exercise on cerebral blood flow. *Med Sci Sports Exerc*. (2015) 47:299–306. doi: 10.1249/MSS.0000000000000422.

The reference for 37 (Chakravarty, 2023) was erroneously written as Chakravarty S. Hydrotherapy and the science of water-based rehabilitation. *J Phys Med Rehabil Stud*. (2023) 5:1–4. doi: 10.47363/JPMRS/2023(5)E111. It should be Chaughule S. Hydrotherapy and the science of water-based rehabilitation. *J Phys Med Rehabil Stud Rep*. (2023) 5:1–4. doi: 10.47363/98z6q539.

The reference for 38 (Lin et al., 2023) was erroneously written as Lin W, Ren F, Chen Z, Li C, Bi Z, Zhang J. Acute effect of breathing exercises on muscle tension and executive function under psychological stress. *Front Psychol*. (2023) 14:1155134. doi: 10.3389/fpsyg.2023.1155134. It should be Liang WM, Xiao J, Ren FF, Chen ZS, Li CR, Bai ZM, et al. Acute effect of breathing exercises on muscle tension and executive function under psychological stress. *Front Psychol*. (2023) 14:1155134. doi: 10.3389/fpsyg.2023.1155134.

The reference for 40 (Wu et al., 2024) was erroneously written as Wu YY, Gao YY, Wang JQ, Zhang C, Xu PJ, Liu J, et al. The influence of mindfulness meditation combined with progressive muscle relaxation training on the clinical efficacy and quality of life of patients with sarcopenia. *BMC Complement Med Ther*. (2024) 24:148. doi: 10.1186/s12906-024-04485-3. It should be Wu YY, Gao YY, Wang JQ, Zhang C, Xu PJ, Liu J, et al. The influence of mindfulness meditation combined with progressive muscle relaxation training on the clinical efficacy and quality of life of patients with sarcopenia receiving haemodialysis: a randomised controlled trial. *BMC Complement Med Ther*. (2024) 24:194. doi: 10.1186/s12906-024-04485-3.

The reference for 44 (George and Kumar, 2025) was erroneously written as George C, Kumar S. Effect of proprioceptive neuromuscular facilitation on balance, strength, cognition and ADL'S in elderly – a randomized control study. *J Bodyw Mov Ther*. (2025) 41:51–7. doi: 10.1016/j.jbmt.2024.10.052. It should be George CM, Kumar S. Effect of proprioceptive neuromuscular facilitation on balance, strength, cognition and ADL'S in elderly – a randomized control study. *J Bodyw Mov Ther*. (2025) 45:1053–7. doi: 10.1016/j.jbmt.2025.10.033.

The reference for 45 (Asadi and Minoonejad, 2023) was erroneously written as Asadi M, Minoonejad H. Effect of eight weeks of Ai chi exercise on balance, proprioception, and performance of elite female athletes with functional ankle instability. *Sci J Rehabil Med*. (2023) 12:504–19. doi: 10.32598/SJRM.12.3.9. It should be Asadi M, Mino-Nejad H, Alizade MH. Effect of eight weeks of Ai Chi exercise on balance, proprioception, and performance of elite female athletes with functional ankle instability. *Sci J Rehabil Med*. (2023) 12:504–19. doi: 10.32598/SJRM.12.3.9.

The reference for 46 (Rana et al., 2025) was erroneously written as Rana S, Dey M, Khandan R. FSR and EMG signal-based gait analysis of Tai-Chi practitioners using Softmax and dropout regularisation. *Multimed Tools Appl*. (2025). doi: 10.1007/s11042-024-20678-x. It should be Rana SP, Dey M, Khandan R. “FSR and EMG signal-based gait analysis of Tai-Chi practitioners using softmax and dropout regularisation”. In: Bhateja V, Patel P, Tang J, editors. *Intelligent Data Engineering and Analytics. Smart Innovation, Systems and Technologies, Vol. 479*. Cham: Springer (2026). p. 250–60. doi: 10.1007/978-3-032-18974-5_21.

The reference for 47 (Law and Li, 2022) was erroneously written as Law N, Li JZ, Khan MA. Biomechanics analysis of seven Tai Chi movements. *Sports Med Health Sci*. (2022) 4:140–6. doi: 10.1016/j.smhs.2022.04.001. It should be Law NY, Li JX. Biomechanics analysis of seven Tai Chi movements. *Sports Med Health Sci*. (2022) 4:245–52. doi: 10.1016/j.smhs.2022.06.002.

The reference for 48 (Mendo et al., 2022) was erroneously written as Mendo B, Gonçalves M, Lopes L, Matos L. Can Yoga, Qigong, and Tai Chi breathing work support the psycho-immune homeostasis during and after the COVID-19 pandemic? A narrative review. *Healthcare (Basel)*. (2022) 10:681. doi: 10.3390/healthcare10040681. It should be Mendo B, Gonçalves M, Lopes L, Matos LC, Machado J. Can Yoga, Qigong, and Tai Chi breathing work support the psycho-immune homeostasis during and after the COVID-19 pandemic? A narrative review. *Healthcare (Basel)*. (2022) 10:1934. doi: 10.3390/healthcare10101934.

The reference for 50 (Qin et al., 2025) was erroneously written as Qin J, Huang J, Liu J, Chen Y, Luo J, Tao L, et al. Effects of Tai Chi Chuan on balance function in adults 60 years or older with type 2 diabetes and mild cognitive impairment in China: a secondary analysis of a randomized controlled trial. *J Diabetes Investig*. (2025) 16:2059–69. doi: 10.1111/jdi.14138. It should be Qin J, Huang J, Liu J, Chen Y, Luo J, Tao L, et al. Effects of Tai Chi Chuan on balance function in adults 60 years or older with type 2 diabetes and mild cognitive impairment in China: a secondary analysis of a multi-center randomized clinical trial. *J Diabetes Investig*. (2025) 16:2059–69. doi: 10.1111/jdi.70138.

Several in-text citation assignments have been corrected. Statements referring to Covill et al. now cite reference 12 rather than reference 25. In Section 3.5, the Parkinson's disease/TUG statement now cites references 19 and 25, and the Tinetti statement cites references 19, 21 and 26. The corresponding Covill-specific citations in the Discussion have been corrected consistently.

Original article:

Section 3.5, Parkinson's disease/TUG: (12, 19) → (19, 25).

Section 3.5, Covill: (25) → (12).

Section 3.5, Tinetti: (12, 19, 21) → (19, 21, 26).

Discussion, Covill/NPRS, trailing citation: (25) → (12).

Discussion, Covill versus Singh: (4, 25) → (4, 12).

Discussion, Covill/Nissim/Oberholster limitations: (20, 21, 25) → (12, 20, 21).

Discussion, Covill/Oberholster further research: (20, 25) → (12, 20).

In addition, the author name “Singh and Sharma” was misspelled in two places and has been corrected to “Singh and Sarma”.

In the Abstract and the Discussion, the reported range of intervention duration was incorrect. It has been corrected to 6 to 14 weeks (1–3 sessions per week), consistent with Section 3.3 and Table 2.

The authors apologize for these errors and state that these corrections do not change the overall interpretation of the findings or the scientific conclusions of the article.

The original version of this article has been updated.

